# Attempt to Infect the Ass with Tuberculin

**Published:** 1899-04

**Authors:** 


### ABSTRACTS FROM FOREIGN JOURNALS.

### GERMAN.

UNDER THE DIRECTION OF J. PRESTON HOSKINS, PH.D.,
PRINCETON, N. J.

ATTEMPT TO INFECT THE ASS WITH TUBERCULIN. By experiments in feeding and injecting, Jolnu came to the following results: The ass is not immune against tuberculosis. The assertion made by Vignerat that the ass resists the subcutaneous injection of tuberculous bacilli is incorrect. Likewise the further assertion that the miliary tuberculosis arising after intravenous injections disappears in the ass, without producing tubercles in the lung, after fifteen to thirty days. Forty-six days after injection typical tubercles as large as a pea, together with other symptoms of acute miliary tuberculosis, were found.—*Thierärzliches Centralblatt*, June 10, 1898.

